# Multiplex PCR methods for detection of several viruses associated with canine respiratory and enteric diseases

**DOI:** 10.1371/journal.pone.0213295

**Published:** 2019-03-04

**Authors:** Xiangqi Hao, Ruohan Liu, Yuwei He, Xiangyu Xiao, Weiqi Xiao, Qingxu Zheng, Xi Lin, Pan Tao, Pei Zhou, Shoujun Li

**Affiliations:** 1 College of Veterinary Medicine, South China Agricultural University, Guangzhou, Guangdong Province, People’s Republic of China; 2 Guangdong Provincial Key Laboratory of Prevention and Control for Severe Clinical Animal Diseases, Guangzhou, Guangdong Province, People’s Republic of China; 3 Guangdong Provincial Pet Engineering Technology Research Center, Guangzhou, Guangdong Province, People’s Republic of China; Nanjing Agricultural University, CHINA

## Abstract

Viral respiratory and intestinal infections are the most common causes of canine viral illness. Infection with multiple pathogens occurs in many cases. Rapid diagnosis of these multiple infections is important for providing timely and effective treatment. To improve diagnosis, in this study, two new multiplex polymerase chain reactions (mPCRs) were developed for simultaneous detection of canine respiratory viruses (CRV) and canine enteric viruses (CEV) using two separate primer mixes. The viruses included canine adenovirus type 2 (CAV-2), canine distemper virus (CDV), canine influenza virus (CIV), canine parainfluenza virus (CPIV), canine circovirus (CanineCV), canine coronavirus (CCoV) and canine parvovirus (CPV). The sensitivity of the mPCR results showed that the detection limit of both mPCR methods was 1×10^4^ viral copies. Twenty nasal swabs (NS) and 20 anal swabs (AS) collected from dogs with symptoms of respiratory disease or enteric disease were evaluated using the novel mPCR methods as a clinical test. The mPCR protocols, when applied to these respiratory specimens and intestinal samples, could detect 7 viruses simultaneously, allowing rapid investigation of CRV (CAV-2, CDV, CIV and CPIV) and CEV (CAV-2, CanineCV, CCoV and CPV) status and prompt evaluation of coinfection. Our study provides an effective and accurate tool for rapid differential diagnosis and epidemiological surveillance in dogs.

## Introduction

Pet dogs play an important role in humans’ daily lives. Recently, the emergence of new pathogens and the continuous circulation of common etiological agents in dog populations have complicated canine diseases [1]. Among these diseases, canine infectious respiratory diseases (CIRD) and viral enteritis pose notable threats to dog health.

CIRD are complex and include canine adenovirus type 2 (CAV-2), canine distemper virus (CDV), canine influenza virus (CIV), canine parainfluenza virus (CPIV), canine herpesvirus (CHV), canine reovirus, *Bordetella bronchiseptica* and other pathogenic agents [2–4]. Among these, CAV-2, CDV or CPIV have frequently been detected in dogs with CIRD, according to previous studies [5, 6]. Avian-origin H3N2 CIV has been detected in domestic dogs in South Korea and China since 2007 [7, 8]. H3N2 CIV is now circulating in dog populations in China, South Korea, Thailand, and even the United States [9–11]. Distinguishing these pathogens can be challenging, because dogs often show similar clinical signs of infection with these viruses, such as low-grade fever, nasal discharge and cough. These respiratory symptoms are flu-like and difficult to diagnose.

Canine viral enteritis is common in dogs with acute vomiting and diarrhea [12]. Canine parvovirus (CPV) is one of the major viruses leading to acute gastroenteritis in dogs; CPV infection is characterized by fever, severe diarrhea and vomiting, with high morbidity [13]. Puppies tend to be intolerant of CPV infection and have higher mortality than adult dogs because of myocarditis and dehydration [14, 15]. Canine coronavirus (CCoV) is characterized by high morbidity and low mortality. Dogs infected with CCoV alone are likely to have mild diarrhea, whereas the disease may be fatal when coinfection by CCoV and CPV, CDV or canine adenovirus type 1 (CAV-1) occurs [16, 17]. CAV-2 is associated with mild respiratory infection and episodic enteritis [18, 19]. Canine circovirus (CanineCV), a newly discovered mammalian circovirus, was first reported by Kapoor et al. in 2012 [20]. CanineCV has been detected in dogs with severe hemorrhagic diarrhea, and it is more common in puppies than in adults [21, 22]. Coinfection of CanineCV with other intestinal pathogens (CPV or CCoV) is closely related to the occurrence of intestinal diseases [23, 24]. Dogs with intestinal diseases are often infected with one or more viruses, and their clinical symptoms are similar [17, 25, 26], making clinical differential diagnosis difficult. To date, no multiplex PCR (mPCR) method has been developed to detect CanineCV and other enteropathogens.

An effective diagnostic tool is important for the prevention, control and treatment of CIRD and viral-enteritis-related viral diseases. Although many methods exist to detect CIRD and canine viral enteritis, most can detect only 2 or 3 pathogens, and the current lack of systematic and comprehensive detection methods makes diagnosis impractical and time consuming [4, 27, 28]. Because mPCR can simultaneously detect multiple pathogens in a timely and inexpensive manner, this technique has become increasingly popular [29]. Therefore, in this study, two new mPCR methods were developed for the detection of canine respiratory viruses (CRV, including CAV-2, CDV, CIV and CPIV) and canine enteric viruses (CEV, including CAV-2, CanineCV, CCoV and CPV), and we indicated that the mPCR methods established here are simple and effective tools for detecting the viruses of interest.

## Materials and methods

### Viruses and bacterial strains

The CAV-2-, CDV-, CPIV- and CPV-positive samples were obtained from the live vaccine Nobivac DHPPi (MSD Animal Health Trading Co., Ltd., Shanghai, China), which contains CAV-2 (10^4.0^ TCID_50_/ml), CDV (10^4.0^ TCID_50_/ml), CPIV (10^5.5^ TCID_50_/ml) and CPV (10^7.0^ TCID_50_/ml). The rabies virus (RABV) was obtained from the inactivated vaccine Nobivac Rabies. The positive CanineCV, CCoV, and H3N2 CIV strains used in this study were maintained in our laboratory. The *Escherichia coli* (*E*. *coli*, ATCC-29522) and *Salmonella enterica* samples were kindly provided by Associate Prof. Zhang, College of Veterinary Medicine, South China Agricultural University.

### Specific primer design

Primer pairs for the detection of CPV and H3N2 CIV were adopted from previous studies [30, 31]. To design primers for viral conserved regions, several sequences of CDV, CPIV, CanineCV and CCoV were compared by multiple alignments using BioEdit Sequence Alignment Editor Version 7.0.9.0 (Ibis Biosciences, Carlsbad, CA, U.S.A.), and primers specific to highly conserved regions of these viruses were designed using Primer Premier 5.0 software (Premier Biosoft International). Primers for the detection of CAV-2 were designed by reference to previous studies with some modification [32]. All primers used for mPCRs were required to have similar annealing temperatures, lack dimmers or hairpin structures, and used degenerate bases as little as possible to avoid mismatches. The primers (Table 1) were synthesized by Tianyihuiyuan Gene Technology Co., Ltd. (Guangzhou, China).

**Table 1:** Primers used for PCR amplification of CRV and CEV.

Virus	Primer name	Primer sequence (5’ to 3’)	PCR products
CAV-2	CAV-2-F^a^	CGCTGARCAYTACTACCTTGTCTATATTTATG	1020 bp
	CAV-2-R^b^	GGTAGAGCWCTTCGTGTCCGCTT	
CDV	CDV-F	AGATTCAGCCATTTGTAGCCA	794 bp
	CDV-R	GTTGGACTACCTGAGCCCTA	
CIV	CIV-F	CAAGCACTAATCAAGAACAAAC	544 bp
	CIV-R	TCTGCTGCTTGTCCTGTACCTT	
CPIV	CPIV-F	ACAAAAATGTCATCCGTGCT	386 bp
	CPIV-R	ATCTCTCCACGGCTCATACC	
CanineCV	CanineCV-F	GCGTTTACCTGTTCACCC	819 bp
	CanineCV-R	AACTGTTTCATCTGCGRCTG	
CCoV	CCoV-F	AGGAAGGCAACAATCCAATA	477 bp
	CCoV-R	GCCACCTCTGATGGACGA	
CPV	CPV-F	AAGACGTGCAAGCGAGTCC	337 bp
	CPV-R	GAGCGAAGATAAGCAGCGTAA	

^a^F, forward primer

^b^R, reverse primer

### Viral nucleic acid extraction and reverse transcription

Total nucleic acids (TNAs) from the positive controls and twenty nasal swabs (NS) and 20 anal swabs (AS) for clinical samples were extracted using the RaPure Viral RNA/DNA Kit (Magen, Guangzhou, China) according to the manufacturer’s protocol. The extracted TNA was divided into two parts; one was used for the amplification of the DNA viruses (CAV-2, CanineCV and CPV) and the other for the reverse transcription (RT) and amplification of the RNA viruses (CDV, CIV, CPIV and CCoV). The RNA extracted from the positive controls and clinical samples was reverse transcribed to cDNA using random primer (TaKaRa, Dalian, China) and Moloney murine leukemia virus (M-MLV) reverse transcriptase (TaKaRa, Dalian, China) according to the manufacturer’s instructions. The cDNA and DNA were stored at -20°C until PCR amplification.

### Standard plasmid preparations

The extracted DNA viruses (CAV-2, CanineCV and CPV) and cDNA of RNA viruses (CDV, CIV, CPIV and CCoV) were used in simplex PCR amplification. The reaction for each virus was performed in a 20-μl volume that contained 10 μl of 2 × Taq Master Mix (Vazyme Biotech, Nanjing, China), 1 μl each of the various templates described above, and 0.5 μl each of the forward primer (10 mM) and reverse primer (10 mM) and 8 μl double distilled water (ddH_2_O). The procedure for simplex PCR was as follows: 95°C for 5 min, followed by 35 cycles of 95°C for 30 s, 57°C for 30 s, and 72°C for 60 s, with a final extension at 72°C for 8 min. The PCR products were evaluated by 1.5% agarose gel electrophoresis.

To construct standard plasmids, the specific PCR products of CAV-2 (1020 bp), CDV (794 bp), CIV (544 bp), CPIV (386 bp), CanineCV (819 bp), CCoV (477 bp) and CPV (337 bp) were ligated into the pMD-18T simple vector (TaKaRa, Dalian, China) after being extracted from the gel using a Universal DNA Purification Kit (Tiangen Biotech, Beijing, China) according to the manufacturer’s instructions. Following transformation, the selected monoclonal bacterial strains were cultured, and the extracted plasmids were verified by PCR.

### Establishment of mPCR methods

The mPCRs were optimized separately for CRV and CEV. Two mixtures containing standard plasmid were used as templates to optimize the annealing temperature (Ta). The primer concentrations used in this study were 10 mM. The reaction for detection of CRV was performed in a 20-μl volume that contained 10 μl of 2× Taq Master Mix, 0.25 μl each of CAV-2-F and CAV-2-R, 0.25 μl each of CDV-F and CDV-R, 0.25 μl each of CIV-F and CIV-R, 0.25 μl each of CPIV-F and CPIV-R, and 40 ng of mixed template (pMD-CAV-2, pMD-CDV, pMD-CIV, pMD-CPIV) and ddH_2_O. The reaction for detection of CEV was also performed in a 20-μl volume; this mixture contained 10 μl of 2 × Taq Master Mix, 0.25 μl each of CAV-2-F and CAV-2-R, 0.5 μl each of CanineCV-F and CanineCV-R, 0.25 μl each of CCoV-F and CCoV-R, 0.25 μl each of CPV-F and CPV-R, 40 ng of mixed template (pMD-CAV-2, pMD-CanineCV, pMD-CCoV, pMD-CPV), and ddH_2_O. Gradient mPCR was performed for each mixture to optimize the reaction with gradient annealing temperatures (Ta) ranging from 55°C to 65°C. Both reaction procedures were as follows: initial denaturation at 97°C for 5 min; 30 cycles of denaturation at 97°C for 30 s, varied Ta for 45 s and 72°C for 1 min; and a final extension at 72°C for 8 min. 10 μl of mPCR product loans were evaluated by 1.5% agarose gel electrophoresis.

### Specificity of mPCR methods

To evaluate the specificity of the mPCRs, we performed specificity assays on CRV and CEV with CRV- and CEV-specific primers, respectively. Similar procedures were used to detect possible cross-reaction of CRV and CEV primers with RNA/DNA extracted from MDCK cells or from other pathogens (RABV, *E*. *coli* and *Salmonella enterica*). The nucleic acid extraction products of the MDCK cells, *E*. *coli* and *Salmonella enterica* were used directly as PCR templates. In contrast, the viral RNA extraction products of RABV required RT prior to use as templates. Both the individual plasmid and premixed plasmids were tested separately in this assay. The empty pMD-18T vector was used as a negative control.

### Sensitivity of mPCR methods

The sensitivity of the mPCR assays was also examined. The concentrations of the seven plasmids (pMD-CAV-2, pMD-CDV, pMD-CIV, pMD-CPIV, pMD-CanineCV, pMD-CCoV and pMD-CPV) were measured using a NanoDrop 2000 UV-vis spectrophotometer (Thermo Scientific, Wilmington, USA). Copy number was calculated by the following formula [33]:

$$\text{copies}/\mu \text{l}=\frac{6\times 10^{23}\left(\text{copies}/\text{mol}\right)\times \text{concentration}\left(\text{g}/\mu \text{l}\right)}{\text{MW}\left(\text{g/mol}\right)}$$

The plasmid mixtures (pMD-CAV-2/pMD-CDV/pMD-CIV/pMD-CPIV for CRV and pMD-CAV-2/pMD-CanineCV/pMD-CCoV/pMD-CPV for CEV) were diluted from 1×10^9^ to 1×10^1^ copies/μl, the calculation method of copy number of individual plasmid was the same as the previous one, and each individual plasmid was diluted from 1×10^10^ to 1×10^1^ copies/μl. Then, the dilutions were used to determine the minimum detection limits of the mPCR methods.

### Reproducibility of mPCR methods

To verify the stability of the assay, the established mPCR methods were performed as three independent mPCR assays by using three different PCR instruments at different times. The plasmid mixtures for CRV and CEV were used as templates after dilution from 1×10^5^ copies/μl to 1×10^3^ copies/μl.

### Evaluation of clinical samples

In the period of February to May 2018, 20 NS and 20 AS were randomly collected from dogs with symptoms of respiratory disease or enteric disease at different animal hospitals in Guangdong Province, China. In this study, NS and AS were selected because they were easy to be collected from animals and can reduce the pain of animals compared with blood samples or other animal samples. Clinical samples were collected with the consent of the animal owners. All swabs were placed in 1 ml ice-cold Dulbecco’s modified Eagle medium (DMEM) containing 10,000 U/ml each of penicillin and streptomycin and centrifuged at 12,000 rpm for 10 min at 4°C to remove impurities. Then, all samples were stored at -80°C until RNA/DNA extraction. Each swab sample was extracted with a volume of 200 μl according to the manufacturer’s protocol. The template was a mixture of 1 μl DNA extracted from each sample and 1 μl cDNA obtained by RT. Finally, each mixed template from the swab samples was subjected to the mPCR methods.

### Ethics statement

All procedures in the sample collection met the requirements and were approved by the Experimental Animal Welfare Ethics Committee of the South China Agricultural University.

## Results

### Establishment of mPCR methods

**Fig 1:**
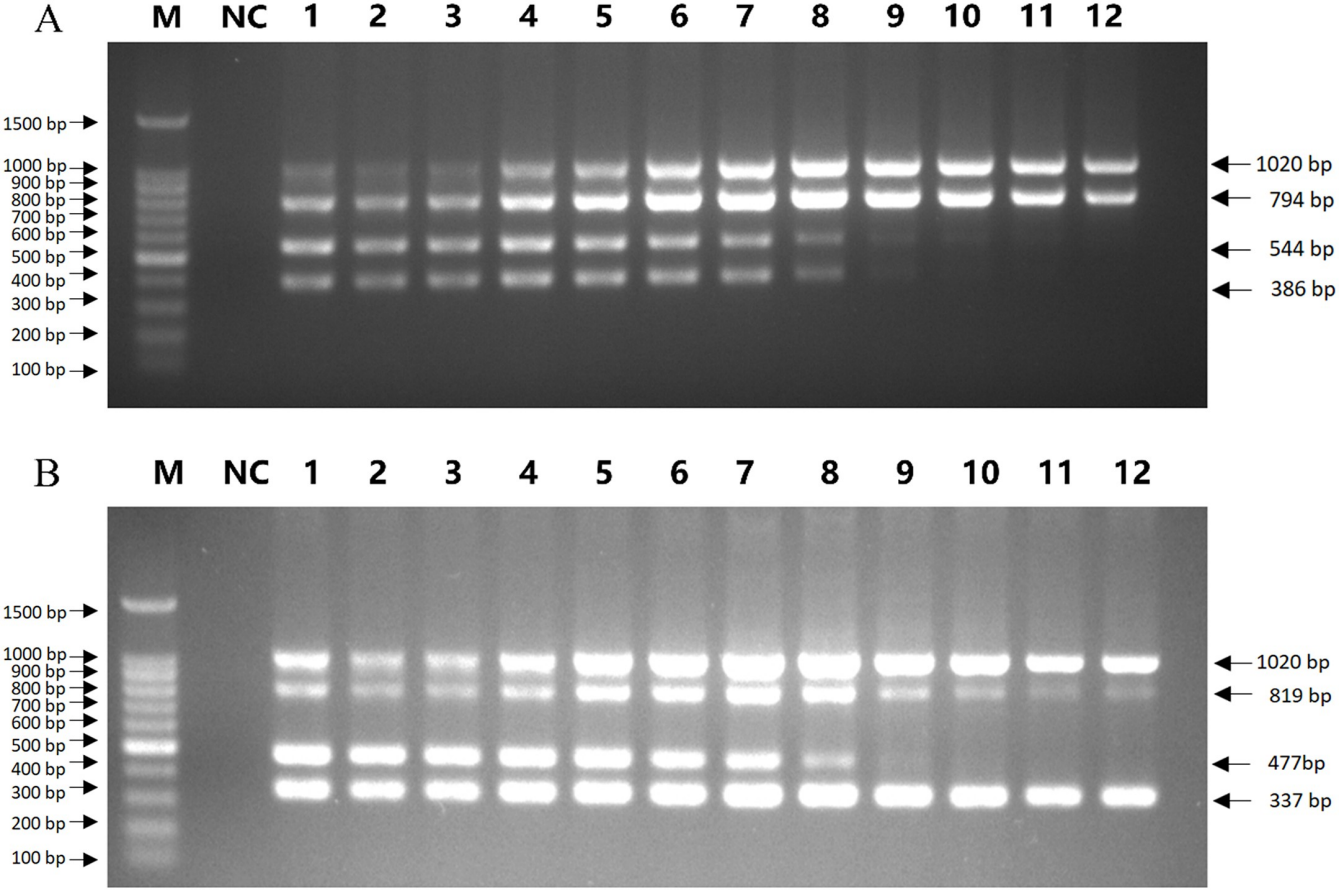
Optimal annealing temperatures in mPCRs. (A) Lane M, 100 bp DNA ladder; Lane NC, negative control for detection of CRV; Lanes 1~12, gradient annealing temperatures were 55.0°C, 55.5°C, 56.0°C, 57.0°C, 58.2°C, 59.4°C, 60.6°C, 61.8°C, 63.0°C, 64.0°C, 64.5°C, and 65.0°C, respectively. (B) Lane M, 100 bp DNA ladder; Lane NC, negative control for detection of CEV; Lanes 1~12, gradient annealing temperatures were 55.0°C, 55.5°C, 56.0°C, 57.0°C, 58.2°C, 59.4°C, 60.6°C, 61.8°C, 63.0°C, 64.0°C, 64.5°C, and 65.0°C, respectively.

The optimal reaction conditions were explored by adjusting the annealing temperature, primer concentration, extension time, and cycle number. Premixed plasmids of CRV and CEV were used as templates to verify the mPCRs, and the products were visualized by 1.5% agarose gel electrophoresis. Ta was evaluated using a gradient, and the optimum Ta values were 59.4°C and 58.2°C, which yielded no primer dimers or nonspecific amplicons for CRV (Fig 1A) or CEV (Fig 1B).

### Specificity of mPCR methods

**Fig 2:**
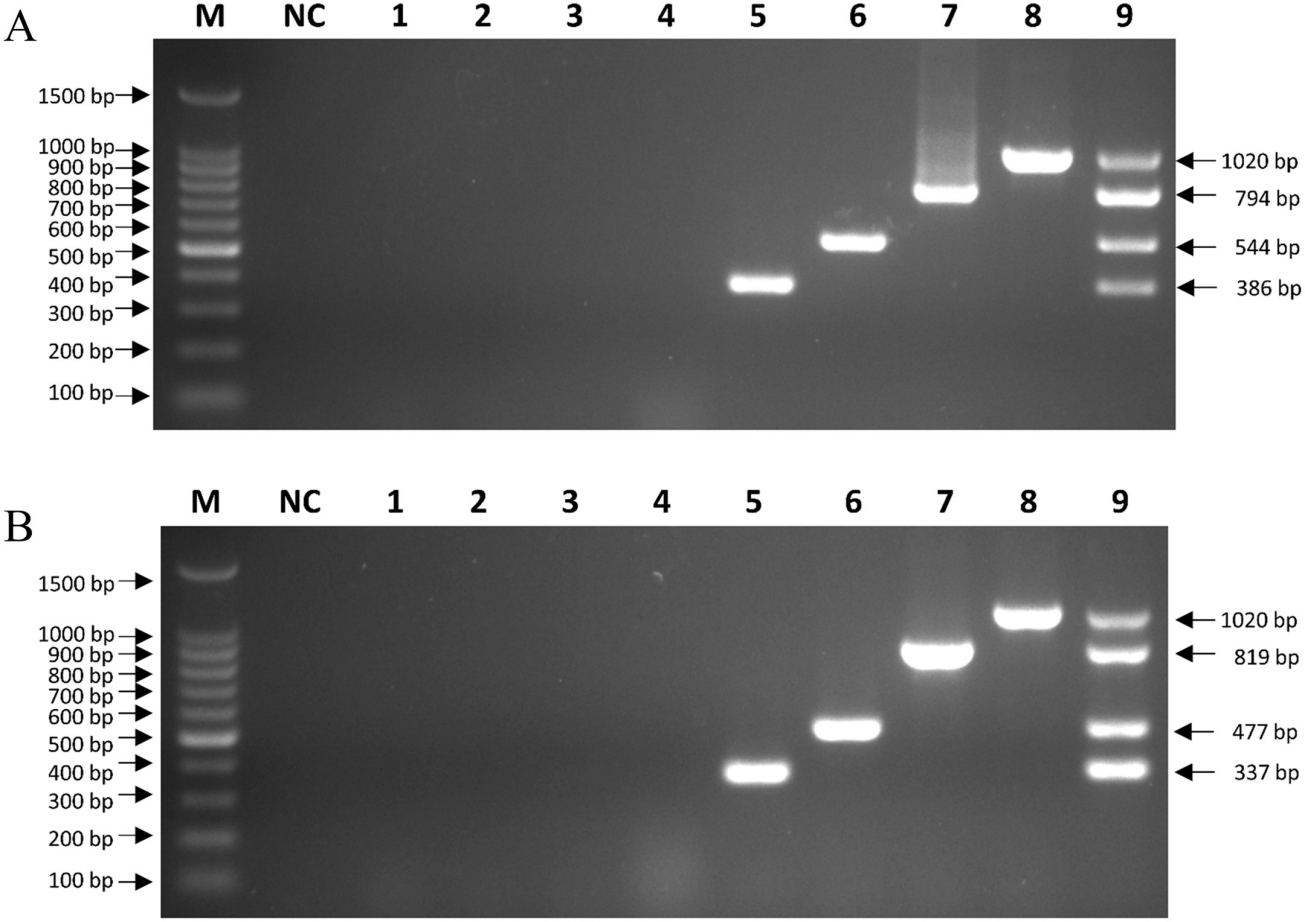
Specificity of the mPCR methods. Agarose gel electrophoresis (1.5%) of specific fragments amplified by mPCRs from the proviral DNAs and cDNA of MDCK cells, *E*. *coli*, *Salmonella enterica* and RABV. (A) CRV mPCR: Lane M, 100 bp DNA ladder; Lane NC, negative control; Lane 1, *E*. *coli*; Lane 2, *Salmonella enterica*; Lane 3, RABV; Lane 4, MDCK cells; Lanes 5~8, pMD-CPIV, pMD-CIV, pMD-CDV, and pMD-CAV-2; Lane 9, mixed standard of pMD-CPIV/pMD-CIV/pMD-CDV/pMD-CAV-2 plasmids. (B) CEV mPCR: Lane M, 100 bp DNA ladder; Lane NC, negative control; Lane 1, *E*. *coli*; Lane 2, *Salmonella enterica*; Lane 3, RABV; Lane 4, MDCK cells; Lanes 5~8, pMD-CPV, pMD-CCoV, pMD-CanineCV, and pMD-CAV-2; Lane 9, mixed standard of pMD-CPV/pMD-CCoV/pMD-CanineCV/pMD-CAV-2 plasmids.

The specificity of the mPCRs was evaluated using other pathogens (RABV, *E*. *coli* and *Salmonella enterica*) and MDCK cells as templates. No specific amplicons showed in the lanes representing negative controls, *E*. *coli*, *Salmonella enterica*, RABV and MDCK cells, whereas the lanes for detection of CRV and CEV showed amplified bands in both CRV mPCR (Fig 2A) and CEV mPCR (Fig 2B).

### Sensitivity of mPCR methods

**Fig 3:**
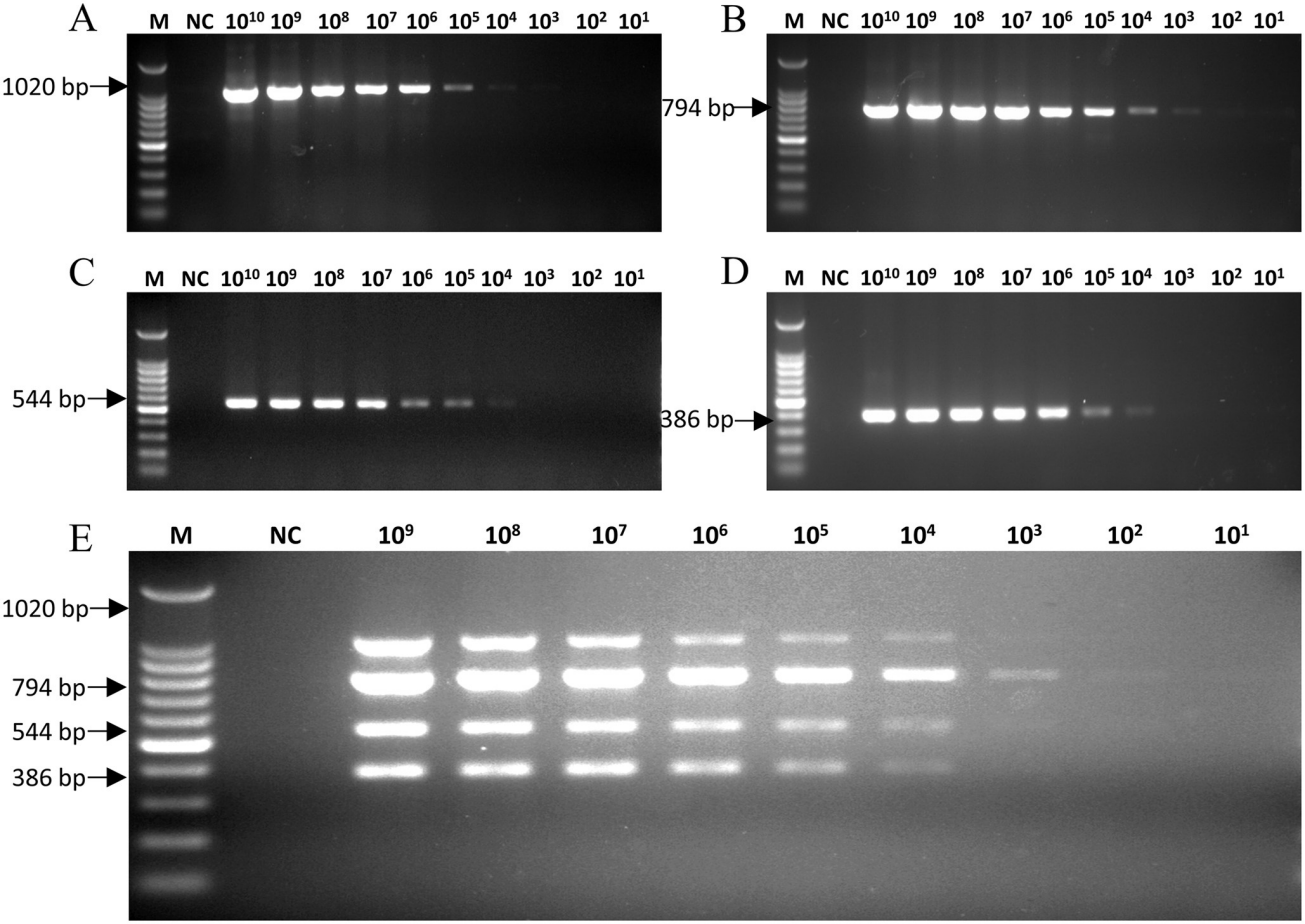
Sensitivity of the mPCR method for CRV detection. The four single plasmids (pMD-CAV-2, pMD-CDV, pMD-CIV and pMD-CPIV), diluted from 1×10^10^ to 1×10^1^ copies/μl, and the mixed plasmids (pMD-CAV-2/pMD-CDV/pMD-CIV/pMD-CPIV), diluted from 1×10^9^ to 1×10^1^ copies/μl, were used to determine the minimum detection limit of the CRV mPCR method. (A) The sensitivity of pMD-CAV-2; (B) the sensitivity of pMD-CDV; (C) the sensitivity of pMD-CIV; (D) the sensitivity of pMD-CPIV; (E) the sensitivity of pMD-CAV-2/pMD-CDV/pMD-CIV/pMD-CPIV; Lane M, 100 bp DNA ladder; Lane NC, negative control.

**Fig 4:**
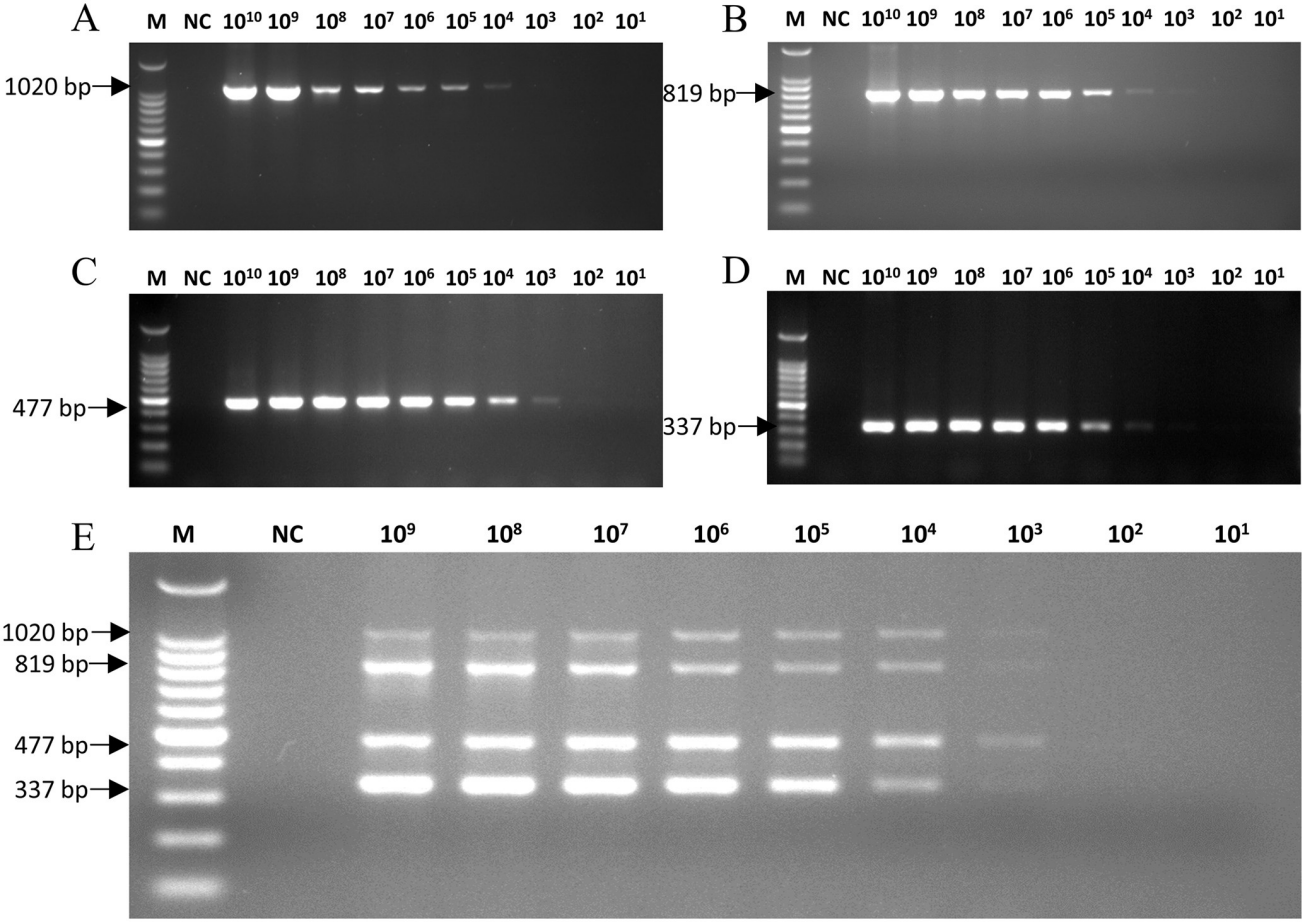
Sensitivity of the mPCR method for CEV detection. The four single plasmids (pMD-CAV-2, pMD-CanineCV, pMD-CCoV and pMD-CPV), diluted from 1×10^10^ to 1×10^1^ copies/μl, and the mixed plasmids (pMD-CAV-2/pMD-CanineCV/pMD-CCoV/pMD-CPV), diluted from 1×10^9^ to 1×10^1^ copies/μl, were used to determine the minimum detection limit of the CEV mPCR method. (A) The sensitivity of pMD-CAV-2; (B) the sensitivity of pMD-CanineCV; (C) the sensitivity of pMD-CCoV; (D) the sensitivity of pMD-CPV; (E) the sensitivity of pMD-CAV-2/pMD-CDV/pMD-CIV/pMD-CPIV; Lane M, 100 bp DNA ladder; Lane NC, negative control.

Each plasmid was used as a template to evaluate the sensitivity of the mPCRs. For CRV, the minimum detection limits for pMD-CAV-2, pMD-CDV, pMD-CIV and pMD-CPIV were 1×10^3^ (Fig 3A), 1×10^3^ (Fig 3B), 1×10^4^ (Fig 3C) and 1×10^4^ (Fig 3D) viral DNA copies, respectively. For CEV, the minimum detection limits for pMD-CAV-2, pMD-CanineCV, pMD-CCoV and pMD-CPV were 1×10^4^ (Fig 4A), 1×10^4^ (Fig 4B), 1×10^3^ (Fig 4C) and 1×10^3^ (Fig 4D) viral DNA copies, respectively. The sensitivity test results revealed that the minimum simultaneous detection limit for mPCR of CRV was 1 × 10^4^ viral copies (Fig 3E), and the limit for CEV (Fig 4E) was also 1×10^4^ viral copies.

### Reproducibility of mPCR methods

**Fig 5:**
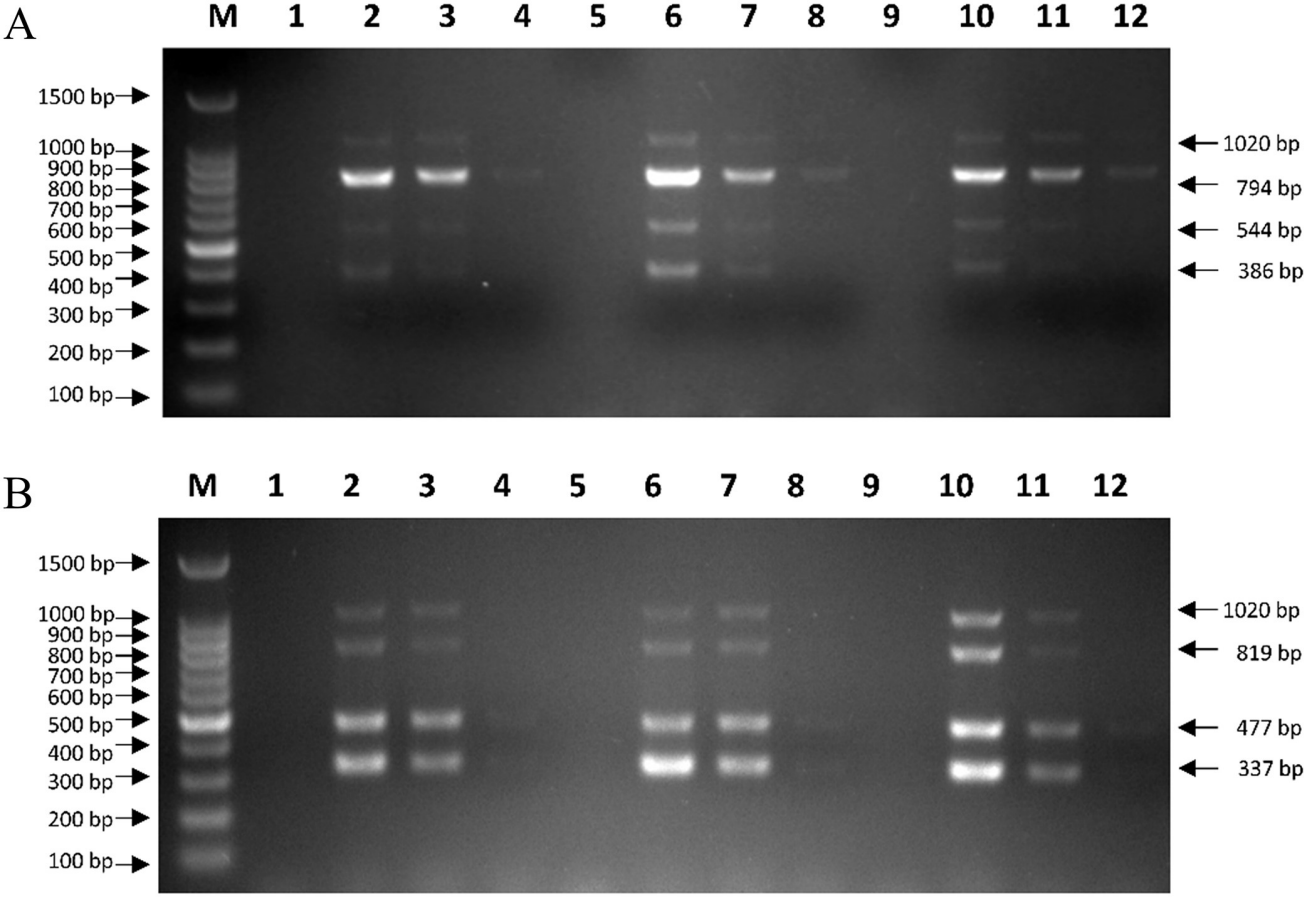
Reproducibility of the mPCR methods. Mixed CRV and CEV plasmids were diluted as templates from 1×10^5^ to 1×10^3^ copies/μl to amplify specific fragments by using three different PCR instruments at different times. (A) CRV: Lane M, 100 bp DNA ladder; Lanes 1, 5, and 9, negative controls for the three tests; Lanes 2~4, mPCR amplifying 1×10^5^ copies/μl, 1×10^4^ copies/μl, and 1×10^3^ copies/μl CRV plasmids; Lanes 6~8, mPCR amplifying different dilutions of mixed plasmids a second time; Lanes 10~12, mPCR amplifying different dilutions of mixed plasmids a third time. (B) CEV: Lane M, 100 bp DNA ladder; Lanes 1, 5, and 9, negative controls for the three tests; Lanes 2~4, mPCR amplifying 1×10^5^ copies/μl, 1×10^4^ copies/μl, and 1×10^3^ copies/μl CEV plasmids; Lanes 6~8, mPCR amplifying different dilutions of mixed plasmids a second time; Lanes 10~12, mPCR amplifying different dilutions of mixed plasmids a third time.

To evaluate the reproducibility of the assay, the detection mPCRs for both CRV and CEV were performed as three independent mPCR assays by using three different PCR instruments at different times. Three premixed plasmids for CRV (Fig 5A) and CEV (Fig 5B), with different dilutions, could be amplified under different conditions and showed similar results among the assays.

### Evaluation of clinical samples

**Fig 6:**
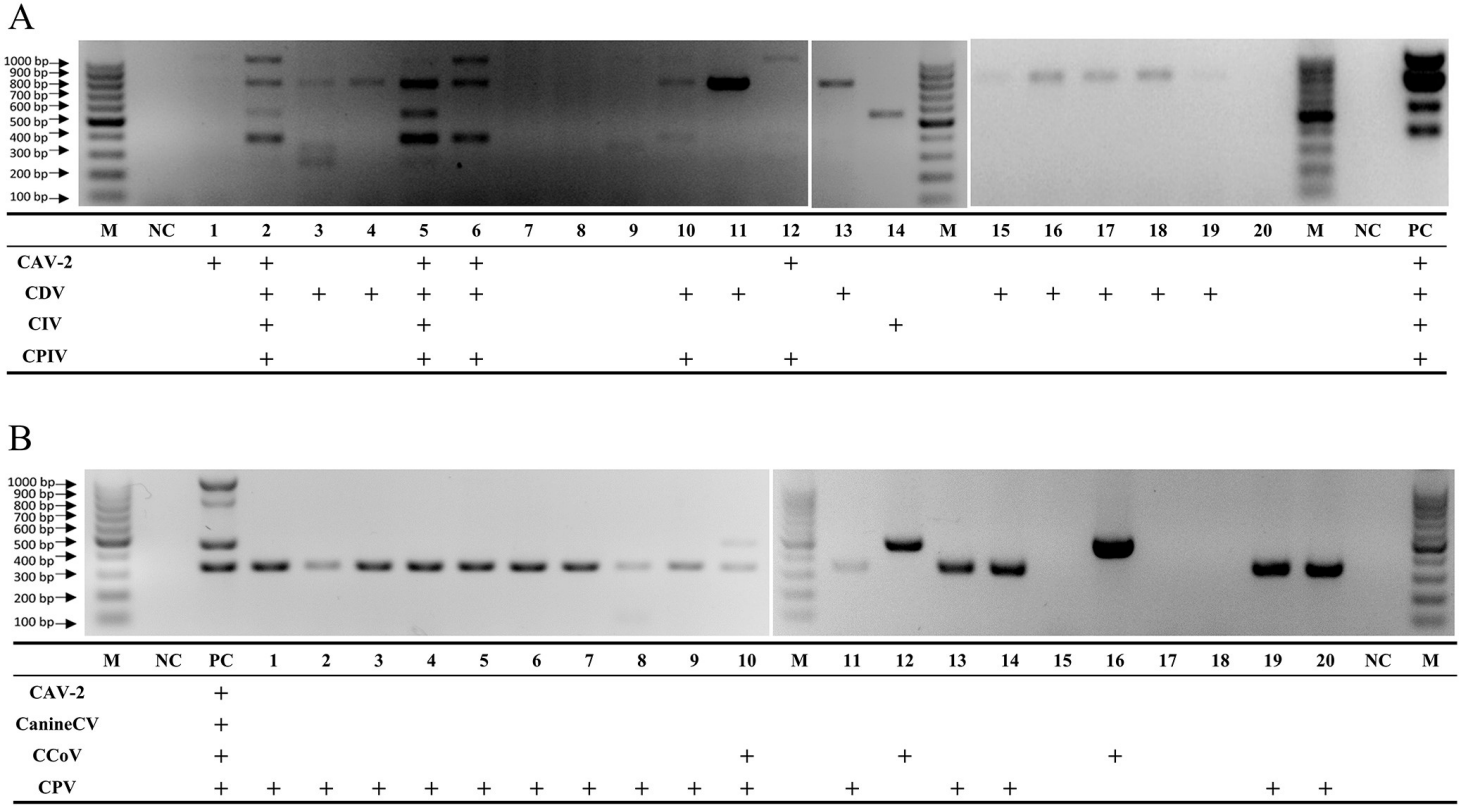
Evaluation of clinical samples. 20 NS and 20 AS clinical samples were tested by established mPCRs respectively. (A) CRV: Lane M, 100 bp DNA ladder; Lanes PC, positive controls; Lanes NC, negative controls; Lanes 1~20, multiplex PCR tested on NS samples. (B) CEV: Lane M, 100 bp DNA ladder; Lanes PC, positive controls; Lanes NC, negative controls; Lanes 1~20, multiplex PCR tested on AS samples.

The mPCRs for detection of CRV (Fig 6A) and CEV (Fig 6B) were tested on 20 NS and 20 AS clinical samples, respectively, and the statistics are shown in Table 2.

**Table 2:** Detection of CRV and CEV in clinical samples using mPCRs.

Target canine respiratory viruses		Target canine enteric viruses	
**Single infection**	**NS** ^(a)^ **(n = 11)**	**Single infection**	**AS** ^(b)^ **(n = 16)**
CAV-2	1	CAV-2	0
CDV	9	CanineCV	0
CIV	1	CCoV	2
CPIV	0	CPV	14
**Dual infection**	**NS (n = 2)**	**Dual infection**	**AS (n = 1)**
CDV+CPIV	1	CCoV+CPV	1
CAV-2+CPIV	1		
**Triple infection**	**NS (n = 1)**	**Triple infection**	**AS (n = 0)**
CAV-2+CDV+CPIV	1		
**Fourfold coinfection**	**NS (n = 2)**	**Fourfold coinfection**	**AS (n = 0)**
CAV-2+CDV+CIV+CPIV	2		

^a)^ NS, nasal swabs;

^b)^ AS, anal swabs; n, number

Detection of CRV in clinical NS samples: 80% (16/20) of the samples were virus positive. Among single infections, CDV was the predominant virus, appearing in 45% (9/20) of NS. Both CAV-2 and CIV were detected in 5% (1/20) of the NS samples. Among dual infections, CDV and CPIV coinfection was identified in 5% (1/20) of NS samples, and CAV-2 and CPIV coinfection was found in 5% (1/20) of NS samples. Only one sample demonstrated triple infection (CAV-2, CPIV and CDV coinfection). Furthermore, two dogs with particularly severe respiratory symptoms were demonstrated to be infected with CAV-2, CDV, CIV and CPIV.

Detection of CEV in clinical AS samples: 85% (17/20) of the samples were virus positive. Among single infections, CPV was the most common virus, appearing in 70% (14/20) of AS, and CCoV was detected in 10% (2/20) of AS samples. Among dual infections, CPV and CCoV coinfection represented 5% (1/20) of AS samples. Notably, CAV-2 and CanineCV were not detected in any of the clinical samples.

## Discussion

At present, the emergence of new pathogens and the continuous circulation of common etiological agents in dogs have made canine diseases more complex and difficult to diagnose. Dog infectious diseases mainly include respiratory and intestinal viral diseases, including CRV (CAV-2, CDV, CIV and CPIV) and CEV (CAV-2, CanineCV, CCoV and CPV). However, the traditional methods of virus identification and isolation are time consuming, causing delays in treatment initiation. A few methods for detecting virus-induced respiratory or enteric disease have been developed [4, 27, 28, 34], but no previous study had developed a systematic way to detect both CRV and CEV in dogs. Here, we developed two mPCR methods for detection of the most frequently coinfected viruses; these methods could be performed to diagnose dogs according to their clinical symptoms.

Primer design is the first and most important step in the process of establishing a detection method, and the following conditions must be satisfied: primers were designed to bind to conserved sequence regions, to have similar annealing temperatures, and to lack dimers or hairpin structures [28]. In these novel mPCR methods, the primer combination produced amplicons that were easy to distinguish from each other, the primer annealing temperatures were similar, and degenerate bases were required only infrequently. The specificity, sensitivity and reproducibility tests all showed good results.

The mPCR methods were tested on 20 NS and 20 AS samples collected from dogs with symptoms of respiratory disease or enteric disease. The ratio of positive samples to total samples was 80% (16/20) for CRV detection and 85% (17/20) for CEV detection. Because the sample number was insufficient, these results were not statistically significant. However, CPV and CDV clearly remain two of the more serious and epidemic diseases in dogs in worldwide at present [35–38]. Epidemiological monitoring of CPV is particularly important because CPV evolves at a rapid rate, similar to that of Porcine Circovirus 3 [39, 40]. Because a small number of dogs were negative for the viruses tested by the CRV or CEV detection assays, although they suffered respiratory illness or intestinal problems, we suggest that some viruses with low prevalence and pathogenic bacteria may also cause disease in dogs [2, 41]. A variety of pathogenic bacteria are often present along with viruses in canine infections [42, 43], and thus, it is essential to expand the coverage of mPCR detection in the future. For example, CIRD also include CHV-1, canine reovirus, and Bordetella bronchiseptica and so on. At the same time, other pathogens causing serious zoonotic diseases, such as pseudorabies virus, should also be monitored in future [44, 45].

In this study, the detection of CanineCV was added to an mPCR method for the first time, because coinfection of this pathogen with other pathogens is common [46]. Though the pathogenic mechanism of CanineCV is unclear, epidemiological testing is important for future research. CanineCV was not detected from the AS clinical samples; perhaps the limited source of these clinical samples was responsible for this result. We didn’t get a lot of clinical samples because it was not easy to get disease samples. CAV-2 mostly replicates in the lower respiratory tract and was detected in the NS samples [2]; however, the CAV-2 primer pair used in this study was probably able to amplify the CAV-1 DNA virus despite the optimization performed [32]. Notably, the live vaccine strains used may have an unavoidable impact on disease detection using the methods developed in this study. Additionally, discriminating between wild-type infections and vaccines is important [34], and therefore, a trend exists toward later development of broad-spectrum and accurate mPCR detection methods. Sometimes, cross contamination may lead to experimental failure. It is worth noting that PCR pretreatment and post-treatmen performed in different isolation zones can effectively avoid pollution. Besides, regular air spray cleaning will also play a role.

In conclusion, these newly established mPCR methods provide an efficient, sensitive, specific and low-cost testing tool for the detection of CRV (CAV-2, CDV, CIV and CPIV) and CEV (CAV-2, CanineCV, CCoV and CPV). The use of Taq Master Mix makes the detection process more convenient and reduces the chance of contamination during the process of sample addition; PCRs can be initiated by simply adding enzyme, ddH_2_O, premixed primers, and template, and thus, this method is superior to other mPCR detection methods. Here, detection of CanineCV was added to mPCR for the first time, making this method suitable for the further study of coinfection by CanineCV and other pathogens. This study provides a novel tool for systematic clinical diagnosis and laboratory epidemiological surveillance of CRV and CEV among dogs.

## References

[1] Pesavento PA, Murphy BG. Common and emerging infectious diseases in the animal shelter. Veterinary pathology. 2014;51(2):478–91. 10.1177/0300985813511129 . 24265288

[2] Buonavoglia C, Martella V. Canine respiratory viruses. Veterinary research. 2007;38(2):355–73. 10.1051/vetres:2006058 . 17296161

[3] Erles K, Dubovi EJ, Brooks HW, Brownlie J. Longitudinal study of viruses associated with canine infectious respiratory disease. J Clin Microbiol. 2004;42(10):4524–9. 10.1128/JCM.42.10.4524-4529.2004 15472304 PMC522361

[4] Jeoung HY, Song DS, Jeong WS, Lee WH, Song JY, An DJ. Simultaneous detection of canine respiratory disease associated viruses by a multiplex reverse transcription-polymerase chain reaction assay. The Journal of veterinary medical science. 2013;75(1):103–6. . 22971595 10.1292/jvms.12-0287

[5] Posuwan N, Payungporn S, Thontiravong A, Kitikoon P, Amonsin A, Poovorawan Y. Prevalence of respiratory viruses isolated from dogs in Thailand during 2008–2009. Asian Biomedicine. 2010;4(4):563–9.

[6] Mitchell JA, Cardwell JM, Leach H, Walker CA, Le PS, Decaro N, et al European surveillance of emerging pathogens associated with canine infectious respiratory disease. Veterinary microbiology. 2017;212:31–8. 10.1016/j.vetmic.2017.10.019 29173585 PMC7117498

[7] Song D, Kang B, Lee C, Jung K, Ha G, Kang D, et al Transmission of avian influenza virus (H3N2) to dogs. Emerging infectious diseases. 2008;14(5):741–6. 10.3201/eid1405.071471 18439355 PMC2600237

[8] Li S, Shi Z, Jiao P, Zhang G, Zhong Z, Tian W, et al Avian-origin H3N2 canine influenza A viruses in Southern China. Infection Genetics & Evolution. 2010;10(8):1286–8. 10.1016/j.meegid.2010.08.010 PMC2950248 20732458

[9] Zhu H, Hughes J, Murcia PR. Origins and Evolutionary Dynamics of H3N2 Canine Influenza Virus. Journal of Virology. 2015;89(10):5406–18. 10.1128/JVI.03395-14 25740996 PMC4442499

[10] Kim JK, Nam JH, Lyoo KS, Moon H, Na W, Song EJ, et al Genetic characterization of an ancestral strain of the avian-origin H3N2 canine influenza virus currently circulating in East Asia. J Microbiol Biotechnol. 2016;26(6):1109–14. 10.4014/jmb.1511.11047 27012241

[11] Voorhees I, Dalziel BD, Glaser A, Dubovi EJ, Murcia PR, Newbury S, et al. Multiple incursions and recurrent epidemic fade-out of H3N2 canine influenza A virus in the United States. 2018. 10.1128/JVI.00323-18 PMC6069211 29875234

[12] Pollock RV, Carmichael LE. Canine viral enteritis. Veterinary Clinics of North America Small Animal Practice. 1983;13(3):551–66. 6316616 10.1016/s0195-5616(83)50059-4

[13] Studdert MJ, Oda C, Riegl CA, Roston RP. Aspects of the diagnosis, pathogenesis and epidemiology of canine parvovirus. Australian Veterinary Journal. 1983;60(7):197 6314962 10.1111/j.1751-0813.1983.tb09581.x PMC7159573

[14] Parrish CR. Pathogenesis of feline panleukopenia virus and canine parvovirus. Baillières Clinical Haematology. 1995;8(1):57–71. 7663051 10.1016/S0950-3536(05)80232-X PMC7134857

[15] Miranda C, Thompson G. Canine parvovirus: the worldwide occurrence of antigenic variants. Journal of General Virology. 2016;97(9):2043–57. 10.1099/jgv.0.000540 27389721

[16] Tennant BJ, Gaskell RM, Kelly DF, Carter SD, Gaskell CJ. Canine coronavirus infection in the dog following oronasal inoculation. Research in Veterinary Science. 1991;51(1):11 1654584 10.1016/0034-5288(91)90023-H PMC7131111

[17] Pratelli A, Martella V, Elia G, Tempesta M, Guarda F, Capucchio MT, et al Severe Enteric Disease in an Animal Shelter Associated with Dual Infections by Canine Adenovirus Type 1 and Canine Coronavirus. Zoonoses & Public Health. 2001;48(5):385–92. 10.1046/j.1439-0450.2001.00466.x PMC7165820 11471849

[18] Hamelin C, Jouvenne P, Assaf R. Association of a type-2 canine adenovirus with an outbreak of diarrhoeal disease among a large dog congregation. J Diarrhoeal Dis Res. 1985;3(2):84–7. 2999217

[19] Macartney L, Cavanagh HM, Spibey N. Isolation of canine adenovirus-2 from the faeces of dogs with enteric disease and its unambiguous typing by restriction endonuclease mapping. Research in Veterinary Science. 1988;44(1):9 2836923

[20] Kapoor A, Dubovi EJ, Henriquezrivera JA, Lipkin WI. Complete genome sequence of the first canine circovirus. Journal of Virology. 2012;86(12):7018 10.1128/JVI.00791-12 22628401 PMC3393582

[21] Decaro N, Martella V, Desario C, Lanave G, Circella E, Cavalli A, et al Genomic characterization of a circovirus associated with fatal hemorrhagic enteritis in dog, Italy. Plos One. 2014;9(8):e105909 10.1371/journal.pone.0105909 25147946 PMC4141843

[22] Dowgier G, Lorusso E, Decaro N, Desario C, Mari V, Lucente MS, et al A molecular survey for selected viral enteropathogens revealed a limited role of Canine circovirus in the development of canine acute gastroenteritis. Veterinary microbiology. 2017;204:54–8. 10.1016/j.vetmic.2017.04.007 28532806 PMC7131434

[23] Cavalli A, Desario C, Kusi I, Mari V, Lorusso E, Cirone F, et al Detection and genetic characterization of Canine parvovirus and Canine coronavirus strains circulating in district of Tirana in Albania. Journal of Veterinary Diagnostic Investigation Official Publication of the American Association of Veterinary Laboratory Diagnosticians Inc. 2014;26(4):563. 10.1177/1040638714538965 24928599

[24] Costa EM, de Castro TX, Bottino FO, Garcia RC. Molecular characterization of canine coronavirus strains circulating in Brazil. Veterinary microbiology. 2014;168(1):8–15. 10.1016/j.vetmic.2013.10.002 24216489 PMC7117457

[25] Kempf C, Schulz BS, Strauch C, Sauterlouis C, Truyen U, Hartmann K. Virus detection, clinical signs, and laboratory findings in dogs with acute hemorrhagic diarrhea: a retrospective study of 935 cases. Tierärztliche Praxis Ausgabe K Kleintiere/heimtiere. 2010;38(2):79 . 22331307

[26] Duijvestijn M, Mughini-Gras L, Schuurman N, Schijf W, Wagenaar J, Egberink H. Enteropathogen infections in canine puppies: (co-)occurrence, clinical relevance and risk factors. Veterinary microbiology. 2016;195:115 10.1016/j.vetmic.2016.09.006 27771056 PMC7130724

[27] Liu D, Liu F, Guo D, Hu X, Li Z, Li Z, et al One-step triplex PCR/RT-PCR to detect canine distemper virus, canine parvovirus, and canine kobuvirus. Journal of Veterinary Medical Science. 2018. 10.1292/jvms.17-0442 PMC6656820 29367517

[28] Deng X, Zhang J, Su J, Liu H, Cong Y, Zhang L, et al A multiplex PCR method for the simultaneous detection of three viruses associated with canine viral enteric infections. Archives of Virology. 2018:1–6. 29675651 10.1007/s00705-018-3828-4 PMC7086948

[29] Elnifro EM, Ashshi AM, Cooper RJ, Klapper PE. Multiplex PCR: optimization and application in diagnostic virology. Clinical Microbiology Reviews. 2000;13(4):559–70. 11023957 10.1128/cmr.13.4.559-570.2000 PMC88949

[30] Lin Ying G Q, Fu Haibin, Li Junhuan, Yan Mingmei, Li Chengyong, Lin Shuming. Establishment and application of duplex PCR for CPV and CCV detection. Progress in Veterinary Medicine. 2011;32(10):71–4 (in Chinese).

[31] Wang C, Wang Q, Hu J, Sun H, Pu J, Liu J, et al A Multiplex RT-PCR Assay for Detection and Differentiation of Avian-Origin Canine H3N2, Equine-Origin H3N8, Human-Origin H3N2, and H1N1/2009 Canine Influenza Viruses. Plos One. 2017;12(1):e0170374 10.1371/journal.pone.0170374 28107507 PMC5249048

[32] Liu Dafei J Y, Qi Ting, Cheng Jing, Liu Likui, Lin Wenjun, Chai Hongliang, Yang Tiankuo, Wang Chong, Hua Yuping, Qu Liandong, Zhang Hongying. Establishment of the multiplex PCR to the detection of CDV, CPV, CAV-I and CAV-II. Chinese Journal of Preventive Veterinary Medicine. 2012;34(11):911–4 (in Chinese).

[33] Wang CY, Hsu CJ, Chen HJ, Chulu JLC, Liu HJ. Development of a reliable assay protocol for identification of diseases (RAPID)-bioactive amplification with probing (BAP) for detection of Newcastle disease virus. Veterinary microbiology. 2008;130(1–2):28–36. 10.1016/j.vetmic.2007.12.015 18261864

[34] Piewbang C, Rungsipipat A, Yong P, Techangamsuwan S. Development and application of multiplex PCR assays for detection of virus-induced respiratory disease complex in dogs. Journal of Veterinary Medical Science. 2016;78(12):1847–54. 10.1292/jvms.16-0342 27628592 PMC5240764

[35] Apaa T, Daly JM, Tarlinton RE. Canine parvovirus (CPV-2) variants circulating in Nigerian dogs. Veterinary Record Open. 2016;3(1). 10.1136/vetreco-2016-000198 PMC5128780 27933190

[36] Zhou P, Zeng W, Zhang X, Li S. The genetic evolution of canine parvovirus–A new perspective. Plos One. 2017;12(3). 10.1371/journal.pone.0175035 PMC5376324 28362831

[37] Li C, Guo D, Wu R, Kong F, Zhai J, Yuan D, et al Molecular surveillance of canine distemper virus in diarrhoetic puppies in northeast China from May 2014 to April 2015. Journal of Veterinary Medical Science. 2018. 10.1292/jvms.17-0559 PMC6021885 29695673

[38] Gowtage-Sequeira S, Banyard AC, Barrett T, Buczkowski H, Funk SM, Cleaveland S. Epidemiology, pathology, and genetic analysis of a canine distemper epidemic in Namibia. Journal of Wildlife Diseases. 2009;45(4):1008 10.7589/0090-3558-45.4.1008 19901377

[39] Li G, He W, Zhu H, Bi Y, Wang R, Xing G, et al Origin, Genetic Diversity, and Evolutionary Dynamics of Novel Porcine Circovirus 3. Adv Sci (Weinh). 2018;5(9):1800275 Epub 2018/09/27. 10.1002/advs.201800275 30250786 PMC6145280

[40] Li G, Wang H, Wang S, Xing G, Zhang C, Zhang W, et al Insights into the genetic and host adaptability of emerging porcine circovirus 3. Virulence. 2018;9(1):1301–13. Epub 2018/07/06. 10.1080/21505594.2018.1492863 29973122 PMC6177243

[41] Jacob J, Lorber B. Diseases Transmitted by Man’s Best Friend: The Dog. Microbiol Spectr. 2015;3(4). 10.1128/microbiolspec.IOL5-0002-2015 26350317

[42] Zheng Y, Hao X, Lin X, Zheng Q, Zhang W, Zhou P, et al Bacterial diversity in the feces of dogs with CPV infection. Microbial Pathogenesis. 2018. 10.1016/j.micpath.2018.04.043 29709688

[43] Sowman HR, Cave NJ, Dunowska M. A survey of canine respiratory pathogens in New Zealand dogs. New Zealand veterinary journal. 2018;66(5):236–42. 10.1080/00480169.2018.1490214 . 29924957

[44] Moreno A, Sozzi E, Grilli G, Gibelli LR, Gelmetti D, Lelli D, et al Detection and molecular analysis of Pseudorabies virus strains isolated from dogs and a wild boar in Italy. Veterinary microbiology. 2015;177(3–4):359–65. Epub 2015/04/29. 10.1016/j.vetmic.2015.04.001 . 25912160

[45] He W, Auclert LZ, Zhai X, Wong G, Zhang C, Zhu H, et al Interspecies transmission, genetic diversity, and evolutionary dynamics of pseudorabies virus. The Journal of infectious diseases. 2018 Epub 2018/12/28. 10.1093/infdis/jiy731 . 30590733

[46] Li L, Mcgraw S, Zhu K, Leutenegger CM, Marks SL, Kubiski S, et al Circovirus in Tissues of Dogs with Vasculitis and Hemorrhage. Emerging infectious diseases. 2013;19(4):534–41. 10.3201/eid1904.121390 23628223 PMC3647419

